# State Adoption of Nursing Home Immunity From Legal Liability During the COVID-19 Pandemic

**DOI:** 10.1093/geroni/igab046.605

**Published:** 2021-12-17

**Authors:** Pamela Nadash, Edward Miller, Elizabeth Simpson, Michael Gusmano, Lisa Beauregard

**Affiliations:** 1 University of Massachusetts Boston, University of Massachusetts Boston, Massachusetts, United States; 2 University of Massachusetts Boston, Boston, Massachusetts, United States; 3 Rutgers University School of Public Health, Rutgers University School of Public Health, New Jersey, United States; 4 Executive Offce of Elder affairs, Executive Office of Elder Affairs, Massachusetts, United States

## Abstract

Twenty-eight states have provided nursing homes (NHs) with immunity from legal liability related to COVID-19. This study places these provisions in the context of prior actions protecting NHs from legal action and explores factors influencing the adoption of such immunity provisions across states. It uses cross-sectional data to examine patterns of policy adoption and to assess states’ likelihood of adopting immunity provisions using multivariate methods. Variables of interest include information on state political, socioeconomic, programmatic, and COVID-19-related characteristics as well as data on campaign contributions and lobbying activity at the state level. Factors significantly related to NH immunity provision adoption included measures of state fiscal health (unemployment), ideology (percent legislators Democrat), governing capacity (unified government), and NH characteristics (percent not-for-profit, hospital-based, and chain). Population density and Medicaid as a percentage of state general fund expenditures proved significant as well. Against these complex influences, organizations lobbying on behalf of NH residents and their families have found themselves ineffectual in creating avenues for accountability. Results indicate that enforcing accountability for NH deaths during the COVID-19 pandemic is a complex process, constrained by available policy tools and made more complicated by factors external to the NH environment that contributed to high death rates. Historically, the NH industry has been successful in avoiding consequences for poor quality care, a pattern that has persisted in that NHs have generally been successful in avoiding liability for negligence during the COVID-19 pandemic.

